# An eleven-year retrospective hospital-based study of epidemiological data regarding human strongyloidiasis in northeast Thailand

**DOI:** 10.1186/s12879-017-2723-z

**Published:** 2017-09-18

**Authors:** Thidarat K. Prasongdee, Pokkamol Laoraksawong, Wanida Kanarkard, Ratthaphol Kraiklang, Kraisit Sathapornworachai, Sureeporn Naonongwai, Porntip Laummaunwai, Oranuch Sanpool, Pewpan M. Intapan, Wanchai Maleewong

**Affiliations:** 1 Department of Parasitology, Faculty of Medicine, Khon Kaen University, Khon Kaen, Thailand; 2 Department of Public Health Administration, Health Promotion, Nutrition, Faculty of Public Health, Khon Kaen University, Khon Kaen, Thailand; 3 Department of Computer Engineering, Faculty of Engineering, Khon Kaen University, Khon Kaen, Thailand; 4 Research and Diagnostic Center for Emerging Infectious Diseases, Mekong Health Science Institute, Khon Kaen University, Khon Kaen, Thailand

**Keywords:** Prevalence, Strongyloidiasis, Hospital-based study, Thailand

## Abstract

**Background:**

Human strongyloidiasis is a chronic and persistent gastrointestinal disease caused by infection with soil-transmitted helminths of the genus *Strongyloides*. The aim of this research was to obtain diagnostic prevalence regarding strongyloidiasis in northeast Thailand through a hospital-based study.

**Methods:**

Patients’ demographic data and the results of stool examinations conducted using the formalin ethyl acetate concentration technique were collected from the parasitology laboratory records at Srinagarind Hospital in Khon Kaen, Thailand. The relevant information from years 2004 to 2014 was collected and descriptively analyzed.

**Results:**

Of a total of 22,338 patients, 3889 (17.4%) had stool samples that tested positive for *Strongyloides* larvae. The highest prevalence was 22.8% (95% CI = 19.6–26.2%) in the year 2004. This percentage progressively decreased, reaching 11.2% (95% CI = 10.2–12.4%) in 2013 and remaining stable at 12.9% (95% CI = 11.8–14.1%) in 2014. Males (2741 cases) had double the positivity rate of females (1148 cases). The prevalence of infection was highest (25.9%; 95% CI = 24.5–27.3%) among patients that were 51–60 years of age.

**Conclusions:**

Areas endemic for strongyloidiasis should be emphasized under the national helminth control program and health education campaigns. Nationwide assessments should also be performed regarding *Strongyloides* infection, including risk factors, treatment, and prevention. The diagnostic laboratory data presented here identify the geographical focus of disease to be the northeastern region of the country. Further targeted surveillance using more sensitive methods will almost certainly reveal a higher individual disease burden than found in this report.

## Background

Human strongyloidiasis is a soil-transmitted helminthiasis caused by two species of the genus *Strongyloides*; *Strongyloides stercoralis* and *Strongyloides fuelleborni* [1, 2]. While *S. fuelleborni*, a zoonotic helminth, is found in Africa and Papua New Guinea, *S. stercoralis* is widespread throughout the world, particularly in Latin America, Southeast Asia, Sub-Saharan Africa, northern Australia, and some parts of the southeastern United States [3].

*Strongyloides stercoralis* has a distinct free-living lifecycle in soil, allowing reproduction and amplification in the direct environment into which an infected person defecates. The disease has spread to areas all over the world, especially tropical and subtropical areas, most likely due to it having been transported with the migration of its human hosts. The worm’s auto-infective life-cycle is the primary reason for its long term persistence in the human host. Thailand is located in the tropical zone and the high-temperatures are one of the risk factors associated with a high prevalence of *S. stercoralis* infection [4]. Studies (both community-based and hospital-based) have shown strongyloidiasis prevalence in Thailand to range from 2.5% to 33.3% [5–9]. The variations in the prevalence data can possibly be attributed to variations in populations and examination methods. Examination methods included direct simple smear, the formalin ethyl acetate concentration technique (FECT), and the agar plate culture (APC) method which exhibit different sensitivity in detecting the infection [5–9]. Furthermore, the hospital-based studies revealed a higher prevalence of *S. stercoralis* infection than the community-based studies. This finding may be attributed to hospital-based studies using stool examination techniques with a higher sensitivity [2].

Srinagarind Hospital is a medical school and tertiary hospital which uses the FECT for parasite examination. Although the FECT has a lower overall sensitivity for the detection of strongyloidiasis than methods such as PCR, agar plate culture, or serology, for clinical purposes, it is sufficient and effective in the diagnosis of symptomatic individuals with larval burdens sufficiently high to be detected by this method [10]. Moreover, as patients who visited Srinagarind Hospital came from different provinces in the northeastern region of Thailand, our hospital-based study may provide insight into the region-wide distribution of strongyloidiasis, as well as other epidemiological data. This paper describes an eleven-year hospital-based study on the diagnostic prevalence of *Strongyloides*-infected patients in Srinagarind Hospital, a tertiary hospital in northeast Thailand.

## Methods

Laboratory records were reviewed of patients seeking medical treatment in Srinagarind Hospital from 2004 to 2014. The routine fecal examination employed in the laboratory was the in-house FECT [11]. While some patients received several FECT examinations due to having visited the hospital multiple times, only one record was analyzed and included in this study. Data on gender, age, residence, and presence of *Strongyloides* larva were collected and analyzed using descriptive statistics with STATA package version 10.1 (StataCorp LLC, College Station, TX).

## Results

**Fig. 1:**
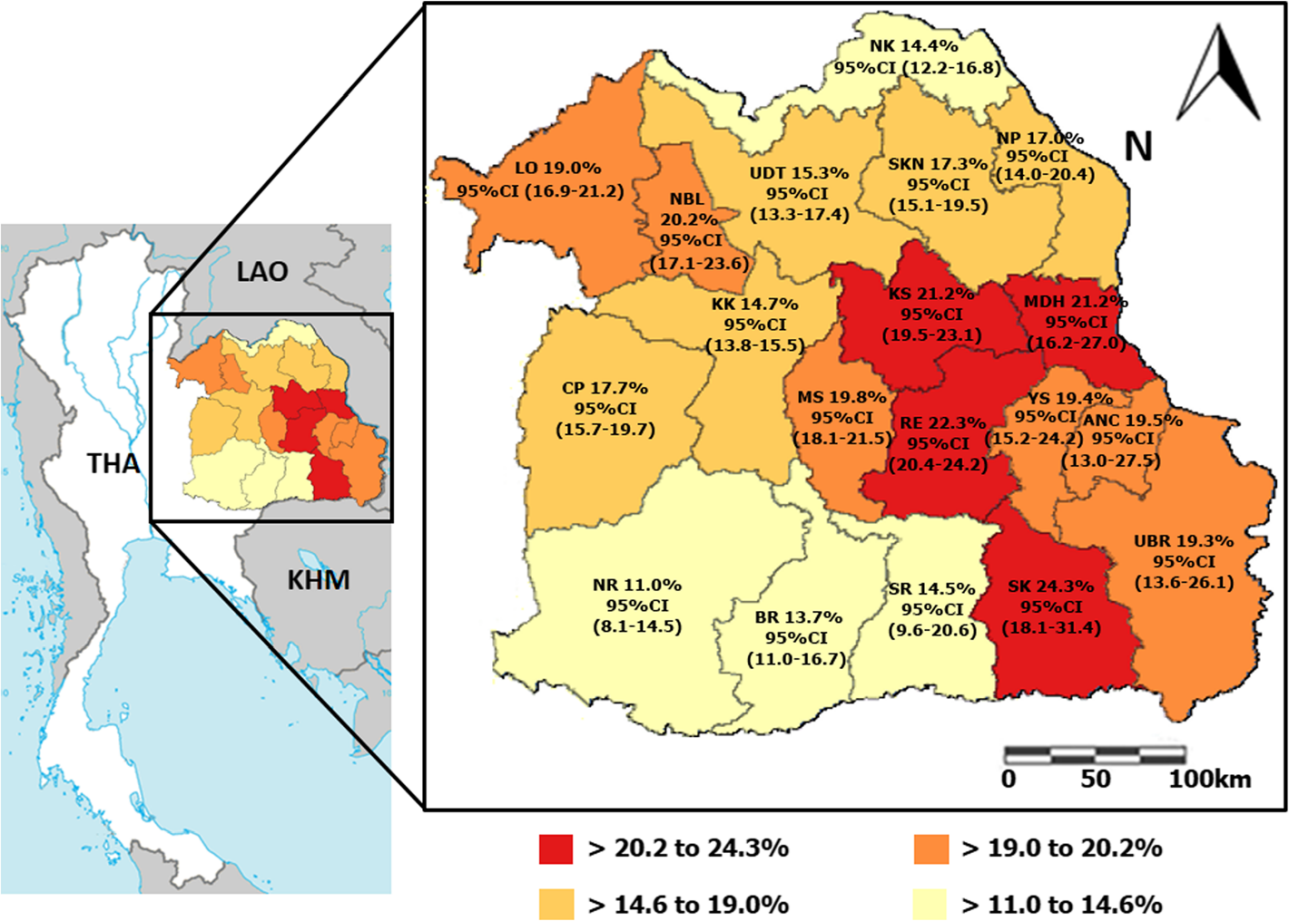
Diagnostic prevalence of *Strongyloides stercoralis* infection by FECT in 19 provinces in northeast Thailand. The map of Thailand was modified from a map in The World Factbook, published by the Central Intelligence Agency [Central Intelligence Agency [US]. The World Factbook is available at: https://www.cia.gov/library/publications/resources/the-world-factbook/geos/th.html Accesed 1 July 2017]. Remark; ANC = Amnat Charoen, BR = Buriram, CP = Chaiyaphum, KS = Kalasin, KK = Khon Kaen, LO = Loei, MS = Maha Sarakham, MDH = Mukdahan, NP = Nakhon Phanom, NR = Nakhon Ratchasima, NBL = Nong Bua Lamphu, NK = Nong Khai, RE = Roi Et, SKN = Sakon Nakhon, SK = Sisaket, SR = =Surin, UBR = Ubon Ratchathani, UDT = Udon Thani and YS = Yasothon. Classified group by quartiles

**Fig. 2:**
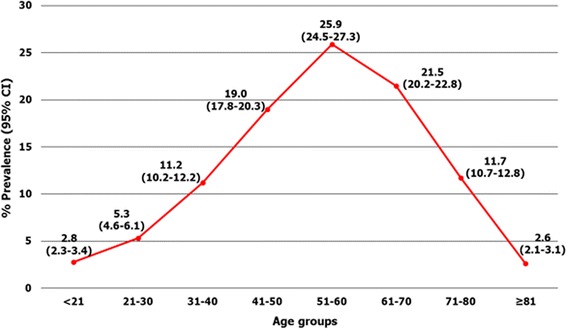
Diagnostic prevalence of *Strongyloides stercoralis* infection classified by age group

A total of 22,338 patient records from 2004 to 2014 were collected. Of these patients, 17.4% (95%CI = 16.9–17.9%) were found to be positive for *S. stercoralis*. The diagnostic prevalence of infection was highest in 2004 at 22.8% (95%CI = 19.6–26.2%) and decreased (with some fluctuation) to 11.2% (95%CI = 10.2–12.4%) in 2013. Prevalence rose slightly in 2014, but the overall downward trend continued to 12.9% (95%CI = 11.8–14.1%; Table 1). In terms of regional distribution among 19 provinces, the diagnostic prevalence was highest in Srisaket province at 24.3% (95%CI = 18.1–31.4%) and lowest in Nakhon Ratchasima province at 11.0% (95%CI = 8.1–14.5%; Fig. 1). In terms of gender, 23.7% (2741/11,589; 95%CI =22.9–24.4%) of *S. stercoralis* positive stool belonged to males and 10.7% (1148/10,749; 95%CI =10.1–11.3%) belonged to females. The higher proportion of infection in males appeared to be constant throughout the 11-year period. The mean age (±SD) of patients was 54.4 (±15.7; range = 3–96) years. Diagnostic prevalence was found to increase with age and reached a peak of 25.9% (95%CI = 24.5–27.3%) among patients who were 51–60 years of age (Fig. 2).

**Table 1:** Number of patients infected with *Strongyliodes stercoralis* and total specimens for diagnosis classified by year^a^

Years	Number of patients infected with *S. stercoralis* (Total specimen for diagnosis)	Diagnostic Prevalence^b^ (95%CI)
2004	144 (632)	22.8 (19.6–26.2)
2005	305 (1361)	22.4 (20.2–24.7)
2006	332 (1723)	19.3 (17.4–21.2)
2007	448 (2000)	22.4 (20.6–24.3)
2008	366 (2122)	17.2 (15.7–18.9)
2009	364 (2283)	15.9 (14.5–17.5)
2010	444 (2519)	17.6 (16.2–19.2)
2011	369 (2762)	13.4 (12.1–14.7)
2012	421 (2891)	14.6 (13.3–15.9)
2013	383 (3405)	11.2 (10.2–12.4)
2014	433 (3346)	12.9 (11.8–14.1)

^a^Number of patients diagnosed by the FECT classified by year some repeated patients included in each studied year

^b^The average diagnostic prevalence was 24.11% which decreased 1.15% each year from 2004 to 2014; Regression equation: Y = 24.11–1.15xi (95%CI = −1.49 to −0.8)

## Discussion

The global prevalence of *S. stercoralis* infection ranges from 10% to 40% [2], and is higher in countries in tropical and subtropical areas with hot and humid climates [2, 12]. Northeast Thailand is located in the equator zone, and the average high temperatures in the cool, rainy, and summer seasons are 30.6 °C, 32.6 °C, and 35.2 °C, respectively [13]. The climate is, thus, congruent with the high prevalence of infection in this area. Previous reports performed in selected communities using different stool examination techniques showed varying prevalence of *S. stercoralis* infection, depending on the sensitivity of the method employed. A direct simple smear, for example, yielded a prevalence of 11.2% [14], while the FECT gave results that ranged from 5.4% to 10.5% [9, 10], and APC results ranged from 15.9% to 28.9% [7–9]. In hospital-based studies in Thailand, diagnostic prevalences have ranged from 2.5% to 17.6% [6, 15]. The diagnostic prevalence of *S. stercoralis* infection in northeast Thailand found in this report also falls within that range at 17.4% (95%CI = 16.9–17.9%). Additionally, Srisaket province was found to have the highest diagnostic prevalence at 24.3% (95%CI = 18.1–31.4%), followed by Roi Et province at 22.3% (95%CI = 20.4–24.2%), Kalasin province at 21.2% (95%CI = 19.5–23.1%) and Mukdahan province at 21.2% (95%CI = 16.2–27.0%). These findings are in agreement with a previous community-based study in which a high prevalence rate was found in Kalasin Province (61.0%) [5]. This could reflect poor personal hygiene in these areas, as inadequate hygienic habits have been shown to be epidemiological factors of strongyloidiasis [12].

In Thailand, the diagnostic prevalence of *S. stercoralis* infection reported in hospital-based studies (34.7%) has been higher than that in community-based studies (23.7%) [2]. This could be due in part to the moderate to high sensitivity of *S. stercoralis* detection techniques performed in hospital laboratories. Moreover, hospital patients tend to be at higher risk and more likely to suffer from underlying diseases including *S. stercoralis* infection [2]. In addition, these patients were able to provide more than one sample for repeated examination. One advantage of a hospital-based study is that patients come from all areas in the region, providing researchers a broad range patient data as well as saving on survey costs compared to community-based studies [2, 16]. Our hospital-based study was, thus, able to provide additional data on the epidemiology of human strongyloidiasis in this area.

The high diagnostic prevalence of strongyloidiasis in this area could also be due to environmental factors, such as lower organic carbon content in the soil and land-use conversion from forest to cropland [17, 18]. The main occupation of people in northeast Thailand is farming, especially rice farming. Land in this region has, thus, been converted from forest to cropland to a greater extent than in other areas [19].

The diagnostic prevalence of human strongyloidiasis in northeast Thailand found in this study means the area can be classified as highly endemic (>5%) [12], but is less so than its neighboring countries, Cambodia and Lao People’s Democratic Republic (Lao PDR) [17, 20, 21]. Northern Cambodia has been shown to have an *S. stercoralis* infection rate of 44.7% [17], and the infection rate in Lao PDR has been reported at 41.0%. Both of these reports were community-based and utilized the APC method [20, 21]. This is not unexpected since the APC method is about four times more sensitive than the FECT [10]. However, if we had performed a true prevalence study in the community using APC rather than a diagnostic prevalence study based on hospital results using the FECT, we may have found a prevalence in northeast Thailand approaching those reported for Laos and Cambodia.

The finding in this study that males were at a higher risk for *S. stercoralis* infection than females corresponded with the results of previous community-based study in Cambodia and a hospital-based study in Thailand [6, 22]. This may be due in part to occupational risks, as a greater proportion of men work in muddy rice fields without footwear, whereas a greater proportion of women work as housewives and wear shoes while walking around in their houses and villages [22]. The direct skin contact with soil that results from walking around barefoot and poor sanitary standards are both risk factors for infective larvae penetration [2]. Interestingly, our study reported the mean age (±SD) of strongyloidiasis patients to be 54.4 ± 15.7, and the highest prevalence of infection (25.9%; 95%CI = 24.5–27.3%) to be in patients 51–60 years of age. This finding is supported by a Brazilian study, which showed that the prevalence of *S. stercoralis* increased with patients’ age, reaching 12.1% among people older than 60 years [12]. In Cambodia, *S. stercoralis* prevalence has also been shown to increase with age, growing exponentially during the first eight years of life, then slowly increasing through remaining years, reaching 51.2% in people 50 years old or older [17]. This is possibly be due to the autoinfective lifecycle, which leads to lifelong infection in almost all patients. Because infection is lifelong, the prevalence of strongyloidiasis incrementally increases with age. This is simply due to the length of time a person has to be exposed to the infection in their environment. The older populations possible have had more time to be exposed and infected. However, the data showed decreasing diagnostic prevalence in patients after they reach the age of 60 (Fig. 2). This result cannot explain, one reason may be the immune systems in most people over the age of 50 with chronic *Strongyloides* infection controlling the infection to the point where larval burden is so low that it cannot be detected by the insensitive FECT method.

## Conclusions

In conclusion, the high prevalence of strongyloidiasis in northeast Thailand found in this study and that of neighboring countries should be emphasized by the national helminth control program and health education campaigns. Nationwide assessments of *S. stercoralis* infection including risk factors, treatment, and prevention should also be conducted. Developing a control strategy is important for the prevention of serious morbidity, which can result from hyperinfection or disseminated strongyloidiasis. However, the data suggest that there has been a gradual decrease in *S. stercoralis* diagnostic prevalence by year (Table 1), which cannot be accurately explained. It is possibly due to the effect of increases in the population’s level of health education.

Nevertheless, due to the limitations of the relatively insensitive FECT and the population that was studied, the diagnostic prevalence data reported here is not completely reflective of the true community prevalence. The people screened in the hospital may represent a selected sub population (i.e. those who can afford hospital care, have gastrointestinal symptoms, and have a high larval load). Moreover, although hospital diagnoses such as those presented here are useful in identifying the comparative prevalence and burden of the disease by district and any foci of infection within districts, it may not reflect the actual prevalence percentages (overall and in each district). These may be shown to differ from (and possibly be higher than) the data reported here if a proper prevalence study using a highly sensitive method and multiple stool examinations per subject were to be performed on a community-wide basis.

## Abbreviations

^°^C: Degree Celsius
95%CI: 95% confidence interval
APC: Agar plate culture
FECT: Formalin ethyl acetate concentration technique
Lao PDR: Lao People’s Democratic Republic
SD: Standard deviation
